# Chemical generation and modification of peptides containing multiple dehydroalanines†
†Electronic supplementary information (ESI) available. See DOI: 10.1039/c5cc05469a



**DOI:** 10.1039/c5cc05469a

**Published:** 2015-07-29

**Authors:** Philip M. Morrison, Patrick J. Foley, Stuart L. Warriner, Michael E. Webb

**Affiliations:** a School of Chemistry and Astbury Centre for Structural Molecular Biology , University of Leeds , Leeds , LS2 9JT , UK . Email: m.e.webb@leeds.ac.uk

## Abstract

Chemical formation of dehydroalanine has been widely used for the post-translational modification of proteins and peptides, however methods to incorporate multiple dehydroalanine residues into a single peptide have not been defined. We report the use of methyl 2,5-dibromovalerate which can be used to cleanly carry out this transformation.

## Abstract 2

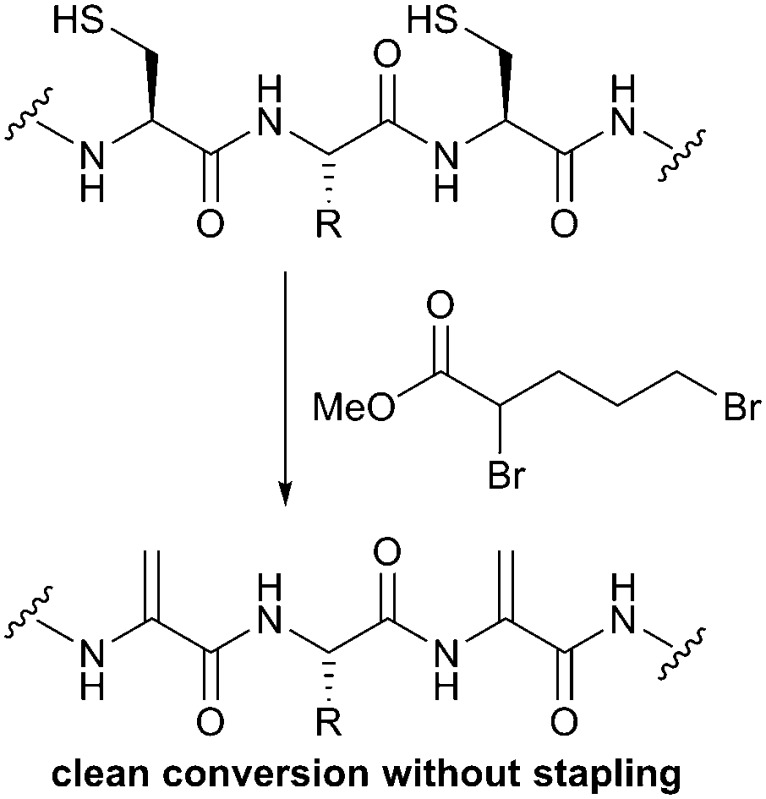
Development of an effective strategy to convert multiple cysteines into dehydroalanine residues within a single peptide using methyl dibromovalerate.

Incorporation of dehydroalanine (Dha) into proteins and peptides can be used to generate a diverse range of chemical modifications. The electrophilic nature of the Dha residue means that it is readily modified in a site-selective fashion using thiols; this has been utilised to label proteins with dyes^1^ and sugars,^2,3^ incorporate unnatural side chains,^4–6^ and install post-translational modifications.^7,8^ A particular advantage of using Dha is that a single modified protein can be reacted with an array of thiols to generate a range of protein conjugates.^5,6^ Metal-catalysed chemistry has also been used to create unnatural amino acids from Dha,^9^ such as those with boron- and silicon-containing side chains^10^ and fluorescent alanine derivatives.^11^ Dha itself is an integral constituent of modified ribosomal peptides such as the lanthipeptides and thiopeptides, being used to generate lanthionine and pyridine cross-links as well as remaining unmodified in many lantibiotic structures. Finally, incorporation of Dha has been used as a chemical biology tool for example as a electrophilic probe in the investigation of diubiquitinase activity and selectivity.^12,13^

Both its application for site-selective protein modification and the involvement of Dha in lanthipeptide biosynthesis have driven the development of methods to chemically generate Dha in peptide structures. Dehydroamino-acids cannot be incorporated using standard peptide synthesis strategies and a masked amino acid is commonly incorporated and subsequently converted to Dha.^14–16^ Biosynthetic methods rely on incorporation of selenocysteine derivatives which are then unmasked using peroxide oxidation^2,17^ whereas enzymatic transformation of serine residues can be achieved utilising dehydratase enzymes from lanthipeptide pathways.^18–23^ All of the chemical methods of Dha generation were reviewed extensively by Davis and coworkers,^24^ who have pioneered the double-alkylation elimination reagents to convert cysteine to Dha. This approach is selective for Cys residues, utilises mild reaction conditions and avoids the incorporation of non-canonical amino acids however it had not been applied to the simultaneous generation of multiple dehydroalanine residues in a single peptide, as has previously only been achieved using unnatural amino acids.^16,25^

Our initial aim was to create constrained peptides in a two-step strategy *via* the creation of multiple Dha residues and their subsequent cyclisation with a small molecule polythiol core (Scheme 1). Similar molecules have been shown to bind to proteins with nanomolar affinities and large surface areas – making them potential modulators of protein–protein interactions.^26–28^ The addition of a thiol to Dha scrambles the stereochemistry at the alpha carbon, often limiting the yield due to formation of the d-Cys derivatives. We aimed to take advantage of the stereochemical scrambling and increase the diversity of peptide loop structures accessible upon cyclisation while incorporating d-amino acids which have previously been shown to improve protease resistance in the bicyclic peptide structures.^29^ Successful application of this strategy was however dependent upon developing a strategy to incorporate the multiple dehydroalanine residues.

**Scheme 1:**
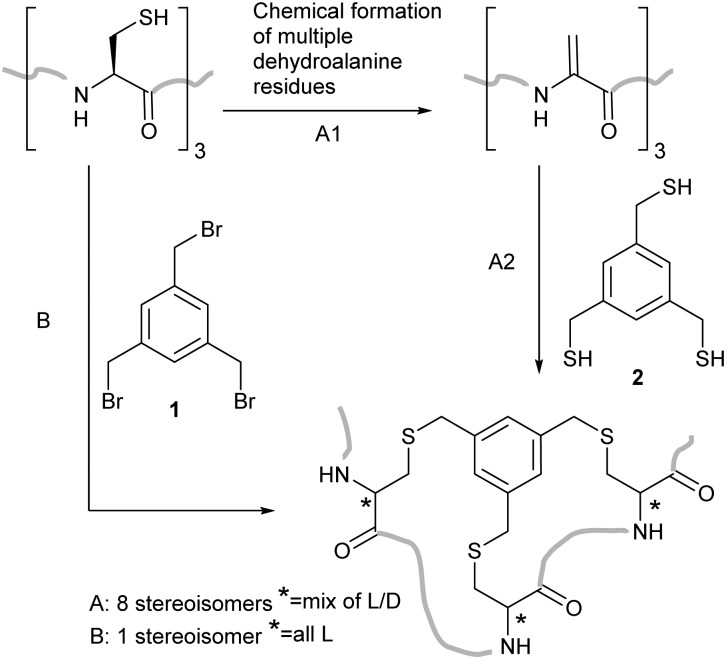
Routes to bicyclic peptide structures involving cysteine modification. (A) A two-step modification in which cysteine is first converted to dehydroalanine followed by cyclisation with a trithiol, ultimately generating 8 stereoisomers of the peptide produced in Route B. (B) Alkylation of 3 cysteine residues with a central mesitylene core.

We planned to utilise cysteine double alkylation for Dha conversion due to the mild reaction conditions and wide tolerance of other amino acids. A cyclic sulfonium intermediate, formed through double cysteine alkylation, is eliminated to generate the dehydroalanine residue.^7,24^ In preliminary experiments with a model peptide containing two cysteines (H_2_N-ACGDDACG-CO_2_H) the use of the most common reagent, dibromoadipamide **3**, led to formation of an undesired stapled by-product (Scheme 2a). While the proportion of the stapled by-product could be decreased by increasing the equivalents of **3** used in the reaction, even at high ratios of reagent to cysteine (50 : 1) residual stapled peptide was still observed. Corresponding experiments with peptides containing three cysteines gave complex mixtures which included a yet higher proportion of stapled peptides.

**Scheme 2:**
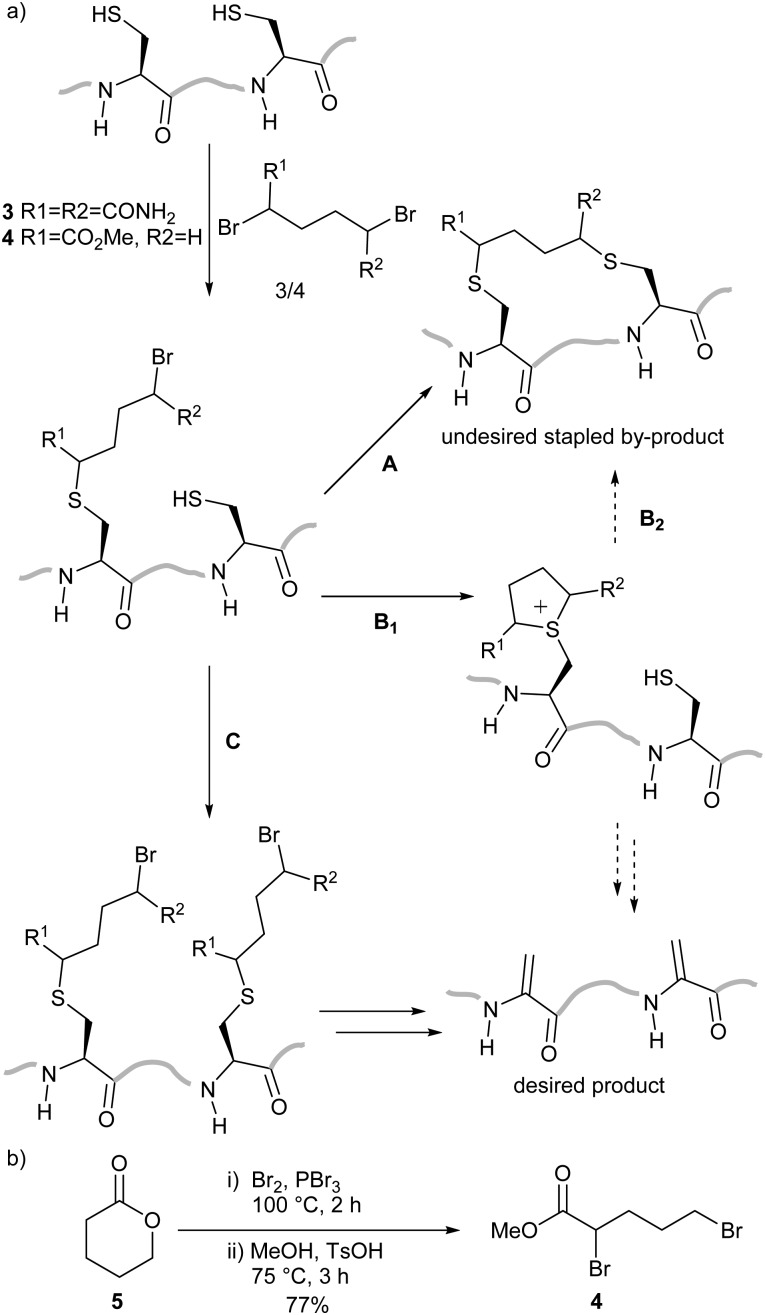
Conversion of multiple cysteine residues to dehydroalanine. (a) The conversion of two cysteine residues with 3 is complicated by formation of a stapled by-product *via* Routes A or B. Stapling is avoided when methyl 2,5-dibromovalerate **4** is used as the lower reactivity of the alkyl bromide slows steps A and B_1_, leading to conversion to dehydroalanine-containing peptides *via* Route C. (b) The synthesis of **4** from δ-valerolactone **5**.

We hypothesised that the stapled by-product forms either by double alkylation of the dibromoadipamide across two cysteine residues (Scheme 2a, Route A), or cysteine interception of the cyclic sulfonium ion (Route B) in a manner similar to elongated thiol modification previously observed by Nathani *et al.* ^30^ An unsymmetrical reagent containing both an α-bromoacyl group and an alkyl bromide would mitigate the observed stapling; the first alkylation step at the α-position would be rapid, whereas a second alkylation at the ω-alkyl bromide would be slow. By changing the relative rates of the two steps we anticipated that all cysteine residues would be monoalkylated before the intramolecular alkylation or sulfonium ion formation, preventing either potential competing reaction pathway (Scheme 2a, Route C).

We therefore generated methyl dibromovalerate **4** *via* PBr_3_-mediated bromination and ring-opening of valerolactone **5** (Scheme 2b). We used **4** to promote alkene formation in the same set of model peptides and observed clean conversion to dehydroalanine residues. Similar conversion was observed for peptides containing three cysteine residues and we were readily-able to purify these peptides by HPLC. To investigate the scope of the reagent, we investigated the reaction of the peptide H_2_N-LTFCEYWAQLCSAA-CO_2_H **6** using both reagents **3** and **4** (Fig. 1a) in a variety of solvent conditions. We monitored by LCMS the formation of both the product peptide **7** containing two Dha residues and the undesired stapled by-products **8** and **9** wherein the two cysteine residues have reacted with a single molecule of **3** and **4** respectively. At low ratios of **4** to **6**, we still observed some formation of the stapled by-product **9** however this was significantly reduced relative to formation of **8** when **3** was used. At higher ratios of **4** to **6** (greater than 5 : 1), little to no stapling could be observed. In DMSO/H_2_O mixtures, no stapling was observed even at low ratios of reagent **4** to peptide **6**. Additionally, the reaction could be conducted in acetonitrile (ESI,† Fig. S1), which is not compatible with **3** since it is not soluble in this solvent. All subsequent conversions were carried out in DMSO/H_2_O followed by HPLC purification.

**Fig. 1:**
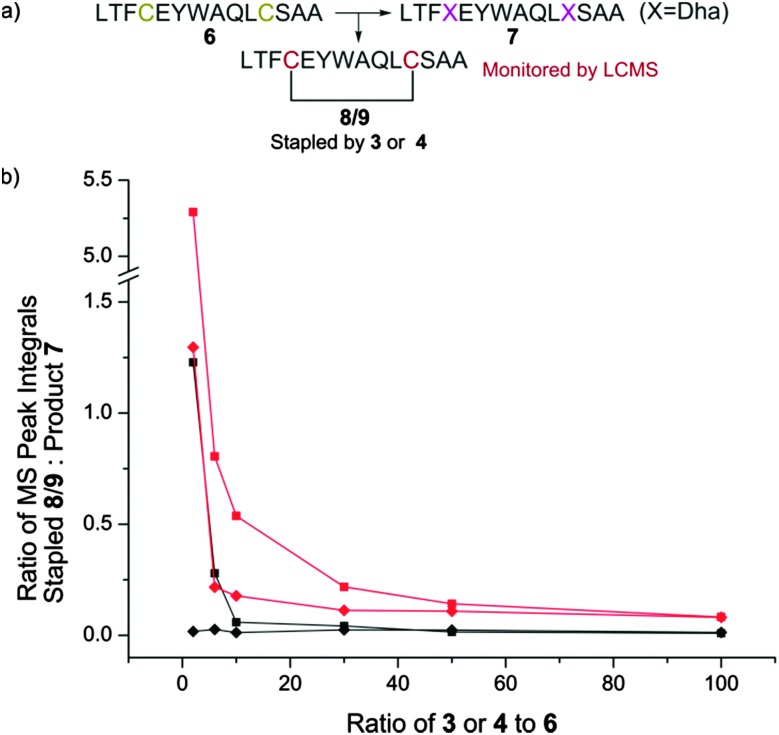
Optimisation of dehydroalanine conversion by LCMS. (a) Peptide **6** was converted to **7** using either **3** or **4**. The level of by-products **8** or **9** were monitored by LCMS when using **3** or **4** respectively. (b) The ratio of stapled by-product **8**/**9** produced in the conversion of cysteine to dehydroalanine in peptide **6** using either **3** or **4** in a variety of base and solvent systems. At low ratios of **3** to **6**, high levels of stapled by-product **8** are observed. This is not entirely mitigated by increasing the equivalents of **3** used, with a persistent residual fraction of stapled peptide observed. The use of **4** demonstrated much reduced staple character, increasing the production of peptide **7** containing multiple dehydroalanine residues. ■ K_2_CO_3_, 1 : 1 H_2_O/DMF; ◆ K_2_CO_3_, 1 : 1 H_2_O/DMSO; red/black = use of **3**/**4** respectively.

Having defined robust chemistry to generate multiple dehydroalanine residues, we turned to the kallikrein inhibitor PK15^28^ as a model system to exemplify the increased diversity generated in our two-step strategy for bicyclic peptide formation. The linear PK15-derived peptide **10** (H_2_N-ACSDRFRNCPADEALCG-CO_2_H) was converted to its dehydroalanine-containing analogue **12** in good yield. To replicate the mesitylene core of PK15 **11** we synthesised the trithiol trismercaptomethylbenzene **2** from tris(bromomethyl)benzene (TBMB) **1**. Addition of **2** to **12** gave a cyclised product as a mixture of stereoisomers (ESI,† Fig. S2).

We were conscious that the bicyclic product has the same mass as monocyclic and uncyclised single-addition products. To confirm the presence of only bicyclic peptide structures, the purified product mixture was individually reacted with either excess *N*-methyl maleimide or β-mercaptoethanol. No reaction of β-mercaptoethanol was observed, and a single addition of *N*-methylmaleimide to the free amino-terminus of the peptide was observed. The same addition was observed on the bicyclic peptide PK15 **11** which was synthesised independently *via* direct cyclisation of peptide **10** with TBMB **1** (Scheme 3).^28^

**Scheme 3:**
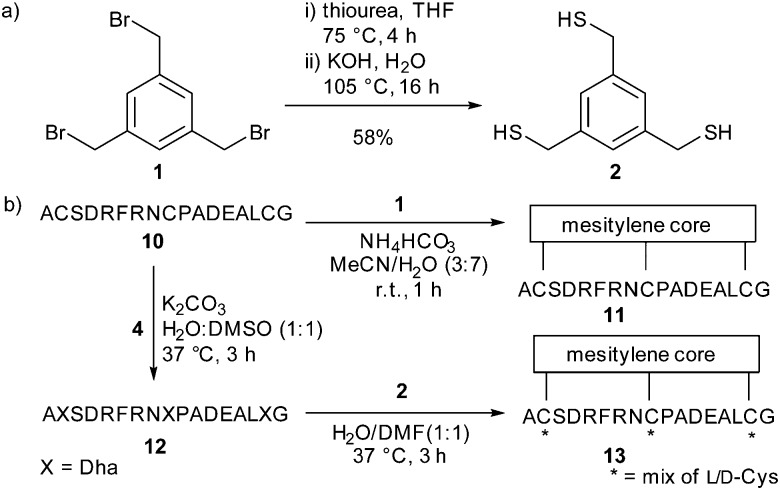
(a) The synthesis of **2** to be used as the central core. (b) The two-step conversion of peptide **10** to the stereochemically-scrambled mixture **13** *via* dehydroalanine-containing peptide **12**.

We evaluated the biological activity of the mixture of stereoisomers **13** obtained *via* cyclisation using a kallikrein inhibition assay. The mixture retained high potency (IC_50_ 144 ± 31 nM), even as a crude mixture before purification (IC_50_ 238 ± 21 nM) (Fig. 2). To further investigate the effect of this stereochemical scrambling on the PK15 sequence we resynthesized the individual stereoisomers using solid phase peptide synthesis and cyclisation with TBMB **1**. Following purification by HPLC, they were then tested in the kallikrein inhibition assay (Table 1 and ESI,† Fig. S3). The range of observed activity spans more than 4 orders of magnitude, albeit with none of the stereoisomers being more potent inhibitors of kallikrein than the literature PK15 LLL-sequence. This differential activity demonstrates that the peptide loops adopt significantly different conformations in response to changes in stereochemistry at a single centre, validating our initial hypothesis that stereochemical scrambling of a peptide library will effectively increase its diversity. Intriguingly, analysis of inhibition by the stereochemically-scrambled peptides revealed an unexpected interaction between the two peptide loops; while the LLD-stereoisomer was inactive in the concentration range tested, a further stereocentre inversion to generate a DLD-variant rescues activity slightly, suggesting that the two loops must interact with each other. The wide range of activities means that we envisage that a deconvolution-by-resynthesis approach could be readily used to identify the most active stereoisomer, if the chemistry were to be adapted to a screening context.

**Fig. 2:**
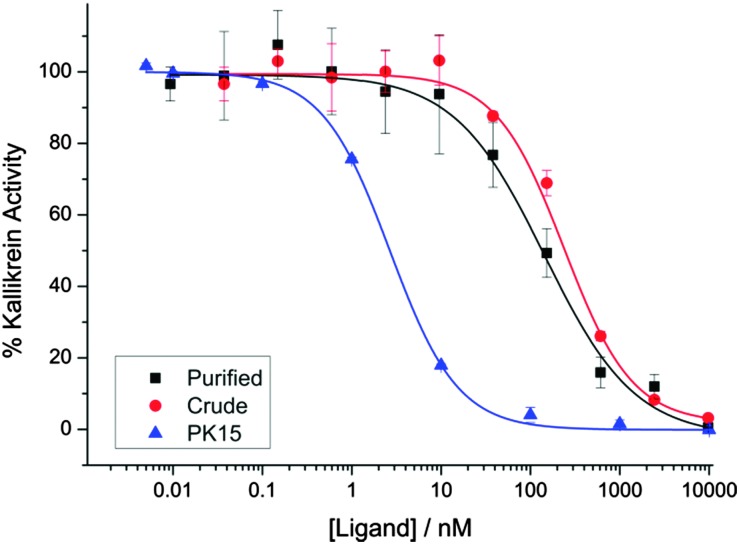
The inhibition of human plasma kallikrein by the mixture of stereoisomers **13**. ▲ PK15 LLL Ligand **8** (IC_50_ 2.7 ± 0.10 nM) ■ purified mixture of isomers **13** (IC_50_ 144 ± 31 nM) crude reaction mixture in the conversion of **12** to **13**. (IC_50_ 238 ± 21 nM).

**Table 1:** IC_50_ data for the 8 stereoisomers of PK15 **11** present in mixture **13**

Isomer	IC_50_/nM	Isomer	IC_50_/nM
LLL^*a*^	2.7 ± 0.10	DDD	>1 × 10^7^
LDD	3740 ± 480	DDL	>1 × 10^7^
LDL	240 ± 14	DLD	1700 ± 50
LLD	>1 × 10^7^	DLL	311 ± 15

^*a*^Naming refers to stereochemical configuration at Cys2, Cys9 and Cys16 in sequence.

In summary, we have reported an effective strategy to generate multiple dehydroalanine residues in peptides *via* mild chemical conversion of cysteines with methyl 2,5-dibromovalerate **4**. This strategy avoids the formation of stapled by-products observed with other reagents. Additionally, **4** is more readily soluble and has a wider solvent tolerance. This reagent has multiple potential applications, including in the synthesis of modified peptides and modified peptide precursors for lanthipeptides. In this report, we have utilised the reagent in a two-step approach to generate stereochemically diverse bicyclic peptide structures from a single peptide precursor. Generation of such mixtures in a peptide or phage-display context and their subsequent deconvolution has the potential to increase the diversity of peptide libraries while simultaneous incorporating in-built resistance to cellular and plasma l-amino- and l-carboxypeptidases as well as increasing the potential range of core molecules which can be used in bicyclic peptide library generation.

We acknowledge support from EPSRC (EP/K03135X/1) for equipment. Personal funding to PMM was provided by the Wellcome Trust (grant 096687/Z/11/Z).
