# Supplementary material for: Chemical generation and modification of peptides containing multiple dehydroalanines
Source: Chem Commun (Camb). 2015 Jul 29;51(70):13470–3. doi: 10.1039/c5cc05469a (PMC4847484; doi:10.1039/c5cc05469a)
Supplement: Supplementary file 1 [file CC-051-C5CC05469A-s001.pdf]

Electronic Supplementary Material (ESI) for ChemComm.
This journal is © The Royal Society of Chemistry 2015

SUPPLEMENTARY RESULTS 2

EXPERIMENTAL METHODS 5

REAGENTS AND EQUIPMENT 5
SYNTHESIS OF 1,3,5-TRIS(MERCAPTOMETHYL)BENZENE (2) 5
SYNTHESIS OF 2,5-DIBROMOADIPAMIDE (3) 5
SYNTHESIS OF METHYL 2,5-DIBROMOVALERATE (4) 6
GENERAL PROCEDURE 1: SOLID PHASE PEPTIDE SYNTHESIS 6
GENERAL PROCEDURE 2: CONVERSION OF CYSTEINE TO DEHYDROALANINE IN PEPTIDES USING 3 6
PEPTIDES 7
LCMS OPTIMISATION OF DEHYDROALANINE FORMATION USING 3 AND 4 7
PEPTIDE CYCLISATION WITH TRIS(BROMOMETHYL)BENZENE (TBMB) TO GENERATE CYCLISED PEPTIDES 11 7
PEPTIDE CYCLISATION WITH TRIS(MERCAPTOMETHYL)BENZENE (TMMB) TO MAKE PEPTIDE MIXTURE 13 8
KALLIKREIN INHIBITION ASSAY 8

NMR DATA 9

Supplementary Results

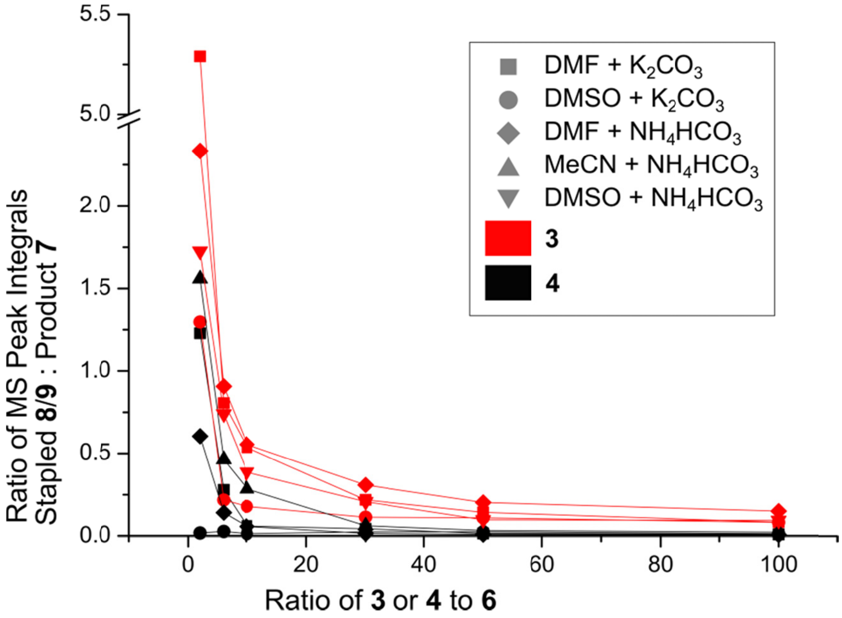

Figure S1: LCMS optimisation of dehydroalanine conversion in peptide **6** using **3** and **4. 4** proved to be superior to **3** in all comparable conditions and was additionally soluble in acetonitrile, allowing examination of the reaction by LCMS without filtration. Conversion using **3** generated stapled by-products at low ratios of **3** to **6**, and even at high ratios a residual fraction of stapled by-product was observed. The use of DMSO and K_2CO_3 with **4** led to extremely low levels of stapled by-product and so these conditions were used in subsequent experiments. Solvents were 1:1 mixture of H_2O and the organic solvent stated in the key. For each reaction condition, the ratio of stapled to dehydroalanine-containing peptide was determined after reaction for 3 hours at 37 °C; this corresponds to complete reaction of the peptide for all except the lowest ratios of **3** and **4** to peptide in which small quantities of residual unmodified peptide were present.

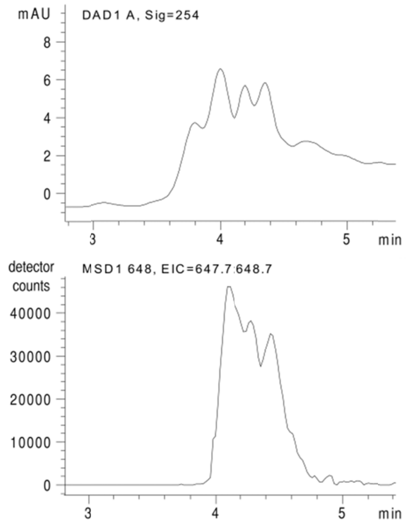

Figure S2: Section of the HPLC purification trace of the peptide H_2N-AXSDRFRNXPADEALXG-CO_2H (X=Dha) showing multiple peaks in the UV trace (top) with mass corresponding to the target peptide (bottom), indicating the presence of multiple stereoisomers.

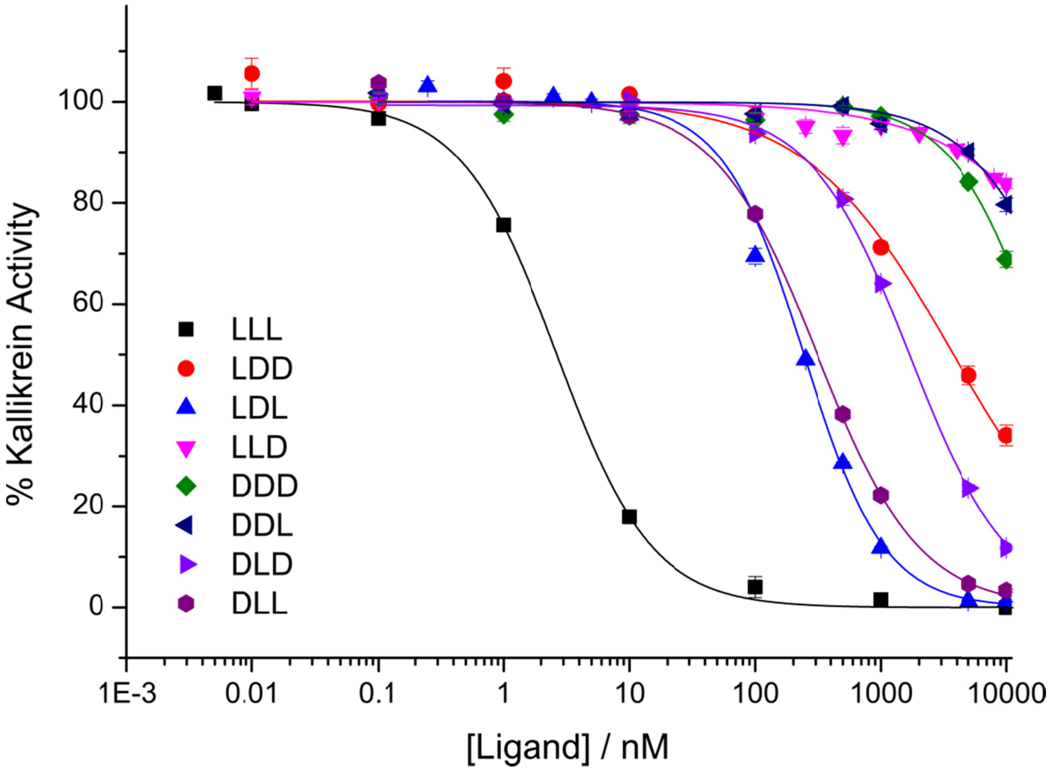

Figure S3: Kallikrein inhibition assay data for individual PK15 stereoisomers. Eight stereoisomers of peptide **10** were synthesised and reacted with **1** to form the PK15 stereoisomer. Ligands are referred to by the stereochemical configurations at Cys2, Cys9 and Cys16 sequentially. Fitted IC_{50} values are given in Table 1.

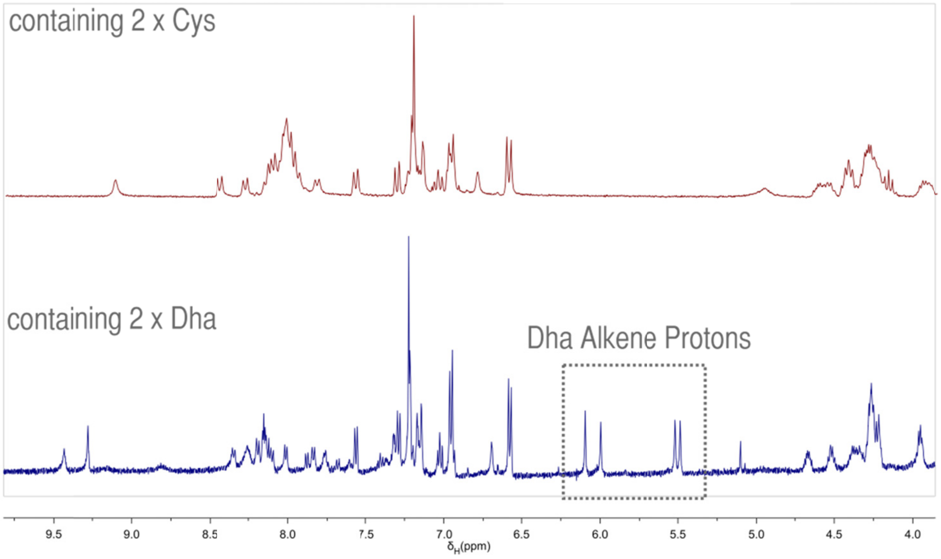

Figure S4: NMR spectra of peptide H_2N-LTFCEYWAQLCSAA-CO_2H (red) and H_2N-LTFXEYWAQLXSAA-CO_2H (X=Dha) with diagnostic alkene protons highlighted.

Experimental Methods

Reagents and Equipment

Unless otherwise stated, all reagents were purchased from Sigma Aldrich, Alfa Aesar, or Fisher Scientific and were used without further purification. Human plasma kallikrein was purchased from Molecular Innovations (HPKA-MIN), Z-Phe-Arg-AMC was purchased from Cambridge Bioscience. NMR data were collected using a Bruker Avance 500, Bruker DRX500, or Bruker DPX300 and analysed using MestReNova software. IR spectra were recorded using a Bruker Platinum-ATR. High resolution mass spectrometry was carried out on a Bruker maXis Impact or on a Bruker Daltonics micrOTOF using electrospray ionisation. Isotopic distributions in routine mass spectra were as expected. Liquid Chromatography Mass Spectrometry (LCMS) was performed on an Agilent 1200 series LC system comprising a Bruker HCT Ultra ion trap mass spectrometer and a Phenomenex Luna C18 50 × 2 mm column (5 μm particle size) using a gradient of 5–90% MeCN over 1.8 min. LCMS data were obtained using a Bruker Ion Trap Mass Spectrometer. Mixtures of solvents, such as those used in column chromatography, are *v/v* and all column chromatography was carried out using Geduran Si 60 silica gel. Unless otherwise stated all non-aqueous reactions were performed under an atmosphere of nitrogen.

Synthesis of 1,3,5-tris(mercaptomethyl)benzene (2)

1,3,5-tris(bromomethyl)benzene (205 mg, 0.575 mmol) and thiourea (230 mg, 3 mmol) were combined and THF (5 mL) added before stirring at 75 °C for 4 h. After cooling to r.t., potassium hydroxide solution (0.68 M, 5 mL) was added and the reaction stirred at 105 °C for 17 h. After cooling to r.t. the reaction mixture was concentrated under reduced pressure before addition of ethyl acetate (20 mL). The mixture was extracted with sodium hydroxide solution (0.1 M, 2 × 20 mL), aqueous layers were combined and acidified (pH 1) with hydrochloric acid (1M) before extraction with ethyl acetate (2 × 30 mL). Organic fractions were combined, dried ($MgSO_4$), filtered and concentrated under reduced pressure. The product was further purified by column chromatography (1:1 ethyl acetate:hexane) to yield **2** (72 mg, 0.33 mmol, 58%) as a colourless oil. δ_H (300 MHz, d_6-DMSO): 7.18 (3H, s, benzene CH), 3.70 (6H, d, J 7.0, benzyl CH_2), 2.84 (3H, t, J 7.0, SH); δ_C (75 MHz, d_6-DMSO): 142.2 (benzene C_q), 126.8 (benzene CH), 28.0 (benzyl CH_2); v_{max}/cm^{-1} (film) 2549 (SH); *m/z* (ES negative): Found [M−H] 215.002092, expected for $[C_9H_{11}S_3]$ 215.002837.

Synthesis of 2,5-dibromoadipamide (3)

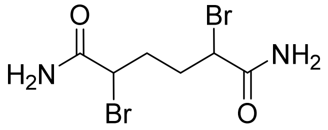

2,5-dibromoadipamide was synthesised following a literature procedure.[1] Adipic acid (12.5 g, 85.6 mmol) was dissolved in $SOCl_2$ (37.5 mL), heated to 80 °C under reflux open to the air for 90 min then cooled to r.t. Carbon tetrachloride (50 mL) was added followed by NBS (34 g, 191 mmol) (NBS freshly recrystallized from H_2O and dried). With vigorous stirring HBr (aq) (48 %, 5 drops) were added and the reaction heated to 80 °C. The reaction turned from red to black over the course of 2.5 h at which point it was cooled to r.t. and then to 0 °C. The precipitated solid was removed by filtration and the flask washed with ether (50 mL). The filtrate was concentrated *in vacuo* to give the crude acid chloride as a dark red viscous liquid.

Ammonium hydroxide solution (25%, 140 mL) was cooled to 0 °C and the crude acid chloride was added dropwise, turning the solution blue. The reaction was stirred for 1 h at 0 °C before the blue solid was isolated by filtration. The solid was suspended in MeOH–H_2O (1:1, 100 mL) and heated to 60 °C for 30 min. Filtration and washing with methanol (100 mL) gave the product **9** (11.36 g, 44%), a mixture of diastereomers, as a grey solid. δ_H (500 MHz, DMSO-d_6): 7.70 (2H, s, NH_2), 7.32 (2H, s, NH_2), 4.37-4.32 (2H, m, BrCH), 2.10-1.79 (4H, m, CH_2-CH_2); *m/z* (ES): Found M+Na^+ 322.900256, expected for [$C_6H_{10}O_2N_2{}^{79}Br_2{}^+$ Na] 322.900122.

Synthesis of methyl 2,5-dibromovalerate (4)

δ-Valerolactone (2.59 g, 25.9 mmol), bromine (1.99 mL, 38.9 mmol) and phosphorus tribromide (47 µL, 0.5 mmol) were combined and the reaction heated at 110 °C for 2.5 h. The reaction was then cooled to 0 °C before the addition of methanol (5.23 mL, 130 mmol) and p-toluenesulfonic acid monohydrate (47 mg, 0.25 mmol). The reaction was heated to 80 °C for 3 h before cooling and removal of excess methanol under reduced pressure. CH_2Cl_2 (50 mL) was added and the lower organic layer obtained, washed sequentially with water (10 mL), NaOH (10%, 10 mL), and water (10 mL). Organics were dried ($MgSO_4$) and concentrated under reduced pressure. Product was further purified by column chromatography (1:4 EtOAc:hexane) to yield **4** (5.457 g, 77%) as a colourless oil. δ_H (300 MHz, $CDCl_3$): 4.27 (1H, dd, J 8.0, 6.0, C2-H), 3.80 (3H, s, methyl), 3.43 (2H, t, J 6.3, C5-H_2), 2.39-1.88 (4H, m, C3-H_2, C4-H_2); δ_C (75 MHz, $CDCl_3$): 169.9 (C1), 53.0 (MeO), 44.5 (C2), 33.3, 32.0 (C5), 30.1; ν_{max}/cm^{-1} (film) 1736 (C=O);

General Procedure 1: Solid Phase Peptide Synthesis

Peptides were synthesised using pre-loaded glycine chlorotrityl resin. During all incubations the tubes were placed on a rotator. Couplings were performed using Fmoc-protected amino acids (5 eq.), HCTU (5 eq.) and DIPEA (5 eq.) with 1 h incubation time at r.t. The resin was washed with DMF (3 × 3 mL, 2 min incubation), with 10% piperidine in DMF (5 × 3 mL, 2 min incubation), DMF (5 × 3 mL, 2 min incubation). After the final coupling reaction the resin was washed with DMF (3 × 3 mL, 2 min incubation), DCM (3 × 3 mL, 2 min incubation) and MeOH (3 × 3 mL, 2 min incubation). The peptide was cleaved and deprotected using a cleavage cocktail (TFA:TIS:EDT:H_2O, 95:1:2.5:2.5, total volume = 3 mL) with incubation for 1 h. The cleavage mixture was eluted into ice-cold ether (40 mL) and the precipitate collected by centrifugation (4000 r.p.m., 5 min). The precipitate was resuspended and washed with ice-cold ether (5 × 15 mL). After drying overnight the peptide was dissolved in water and lyophilised to yield final product.

General Procedure 2: Conversion of Cysteine to Dehydroalanine in Peptides using 3

The peptide was dissolved in water (0.4 mL) and TCEP (0.5 eq.) added. A solution of 2,5-dibromoadipamide (10 eq.) in DMF (0.1 mL) was added before the entire solution was transferred to a vial containing K_2CO_3 (5 eq.). The vial was incubated in a 37 °C shaking incubator at 140 rpm. Reaction was monitored by LCMS.

Peptides

H_2N-AYCDG-CO_2H

Synthesised using general procedure 1. *m/z* (ES): Found $M+H^+$ 528.175738, expected for [$C_{21}H_{29}O_9N_5S$ + H] 528.175875. HPLC: 1.56 min (5-95% Ascentis Peptide Gradient).

H_2N-ACGDDACG-CO_2H

Synthesised using general procedure 1. *m/z* (ES): Found $M+Na^+$ 733.190252, expected for [$C_{24}H_{38}O_{13}N_8S_2$ + Na] 733.189196. HPLC: 4.76 min (0-5% Ascentis Peptide Gradient).

H_2N-LTFCEYWAQLCSAA-CO_2H (6)

Purchased from Thermo Scientific (>80% purity)

H_2N-ACSDRFRNCPADEALCG-$CONH_2$ (8 stereoisomers) (10)

Synthesised using general procedure 1. *m/z* (ES): Found $M+2H^+$ 913.890594, expected for [$C_{72}H_{117}N_{25}O_{25}S_3$] 913.890180.

H_2N-AXSDRFRNXPADEALXG-$CONH_2$ (X=Dha) (12)

Crude peptide H_2N-ACSDRFRNCPADEALCF-CO_2H **7** (10 mg, 5.5 µmol) was incubated with TCEP (0.7 mg) in H_2O (4.4 mL) for 45 min before addition of methyl 2,5-dibromovalerate (90 mg, 328 µmol) in DMSO (4.4 mL) and K_2CO_3 (113 mg, 821 µmol). The reaction was incubated at 37 °C for 3 h before purification by mass-directed HPLC (5-50% MeCN:H2O gradient + 0.1% formic acid). Product-containing fractions were combined, concentrated under reduced pressure before removal of water by lyophilisation to yield pure product **9** as a white flocculent solid (3.5 mg, 37%). *m/z* (ES): Found $M+2H^+$ 862.908689, expected for [$C_{72}H_{111}O_{25}N_{25}$] 862.908599.

LCMS optimisation of dehydroalanine formation using 3 and 4

Peptide (1.2 mg, 747 nmol) was pretreated with TCEP (0.09 mg, 373.5 nmol, 0.5 eq.) in water (300 µL), after half an hour at 37 °C this solution was used as the peptide stock solution. In a 96-well plate (polypropylene, Greiner Bio-one 651201), reactions were set up with the following conditions: peptide **6** 0.623 mM, base (K_2CO_3 or NH_4HCO_3, 62.3 mM), dibromo compound (**3** or **4**, ranging from 1.2 mM to 62.3 mM), 1:1 water:organic (DMF/DMSO/MeCN) total volume 20 µL. After incubation at 37 °C for 3 hours, the reactions were examined using LCMS with blank runs as spacers between samples. Ions corresponding to the product **7** and stapled by-products **8**/**9** were extracted using Bruker ESI Compass data analysis software and the extracted ion peaks integrated. The ratio of these MS peak integrals of **7** to **8**/**9** was plotted to estimate levels of both product and stapled by-product.

Peptide Cyclisation with tris(bromomethyl)benzene (TBMB) to generate cyclised peptides 11

Cyclisation reaction was carried out using the protocol in Heinis et al.[2] Crude peptides (1.83 mg, 1 µmol) were dissolved in 70% (v/v) ammonium bicarbonate (20 mmol, 0.3 mL, pH 8) 30% acetonitrile (0.3 mL) with TBMB (0.36 mg, 1 µmol) added and agitated for an hour. The cyclised peptides were then purified using mass directed HPLC (5-95% MeCN + 0.1 % formic acid, 8 min) to give: PK15 (0.58 mg, 0.29 µmol, 29%); LLD (0.19 mg, 0.10 µmol, 10%); LDL (0.32 mg, 0.16 µmol, 16%); LDD (0.46 mg, 0.24 µmol, 24%); DDD (0.50 g, 0.257 µmol, 26%); DLL (0.50 g, 0.257 µmol, 26%); DLD (0.3 mg, 0.155 µmol, 16%); DDL (0.5 mg, 0.257 µmol, 26%).

Peptide Cyclisation with tris(mercaptomethyl)benzene (TMMB) to make peptide mixture 13

Peptide **12** (2 mg, 1.15 µmol) combined with 1,3,5-tris(mercaptomethyl)benzene **1** (0.3 mg, 1.39 µmol) and K_2CO_3 (1.6 mg, 11.59 µmol) in 1:1 H_2O:DMF (1 mL). The reaction was incubated at 37 °C for 3 h before purification by mass-directed HPLC. Product-containing fractions were combined, concentrated under reduced pressure before removal of water by lyophilisation to yield pure product **13** as a white solid (1.1 mg, 43%) A quarter of the reaction mixture was kept crude for testing in the kallikrein inhibition assay. *m/z* (ES): Found $M+2H^+$ 970.910013, expected for [$C_{81}H_{123}O_{25}N_{25}S_3$] 970.913655.

Kallikrein Inhibition Assay

The inhibitory activity (IC_{50}) of each bicyclic peptide was determined by measuring the residual activity of HPK upon incubation for 30 min at room temperature with concentrations of bicyclic peptide ranging from 10 µM to 0.05 nM. The activity of HPK (2×10^{-4} µM) was measured using fluorogenic substrate Z-Phe-Arg-AMC (0.1 µM) in Tris (10 mM, pH 7.4) supplemented with NaCl (150 mM), $MgCl_2$ (10 mM), $CaCl_2$ (1 mM), BSA (0.1%, *w*/*v*), Triton X-100 (0.01%, *v*/*v*) and DMSO (5%, *v*/*v*). Excitation at 355 nm, emission 460 nm. Measurements were taken every 30 seconds. A linear fit of the data points from 1.5 min to 30 min gave the rate of substrate cleavage. These were then converted to percentage kallikrein activity and plotted against the inhibitor concentration. A logistic fit was used to determine the IC_{50}.

NMR Data

Methyl 2,5-dibromovalerate (**4**)

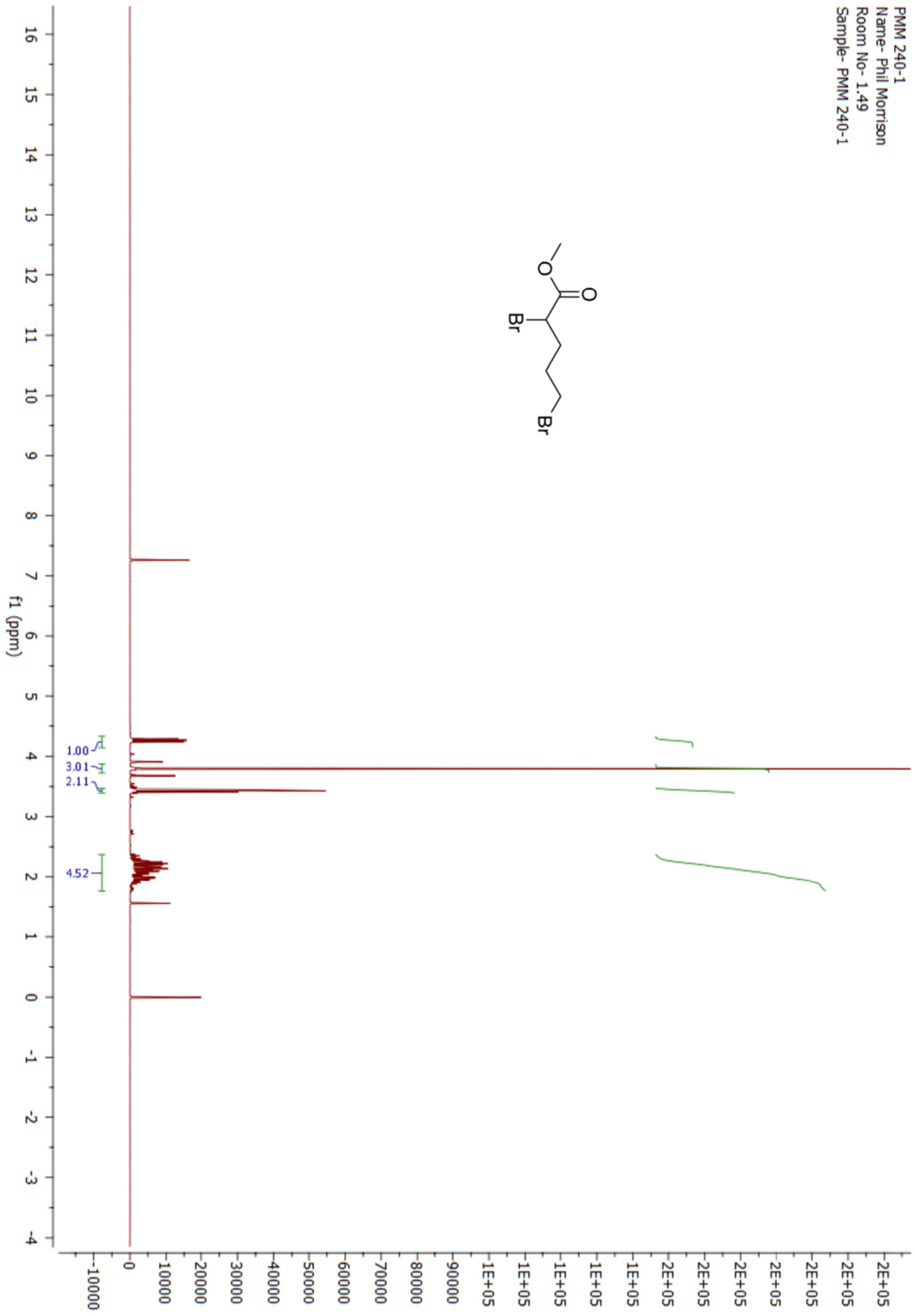

1,3,5-tris(mercaptomethyl)benzene (**2**)

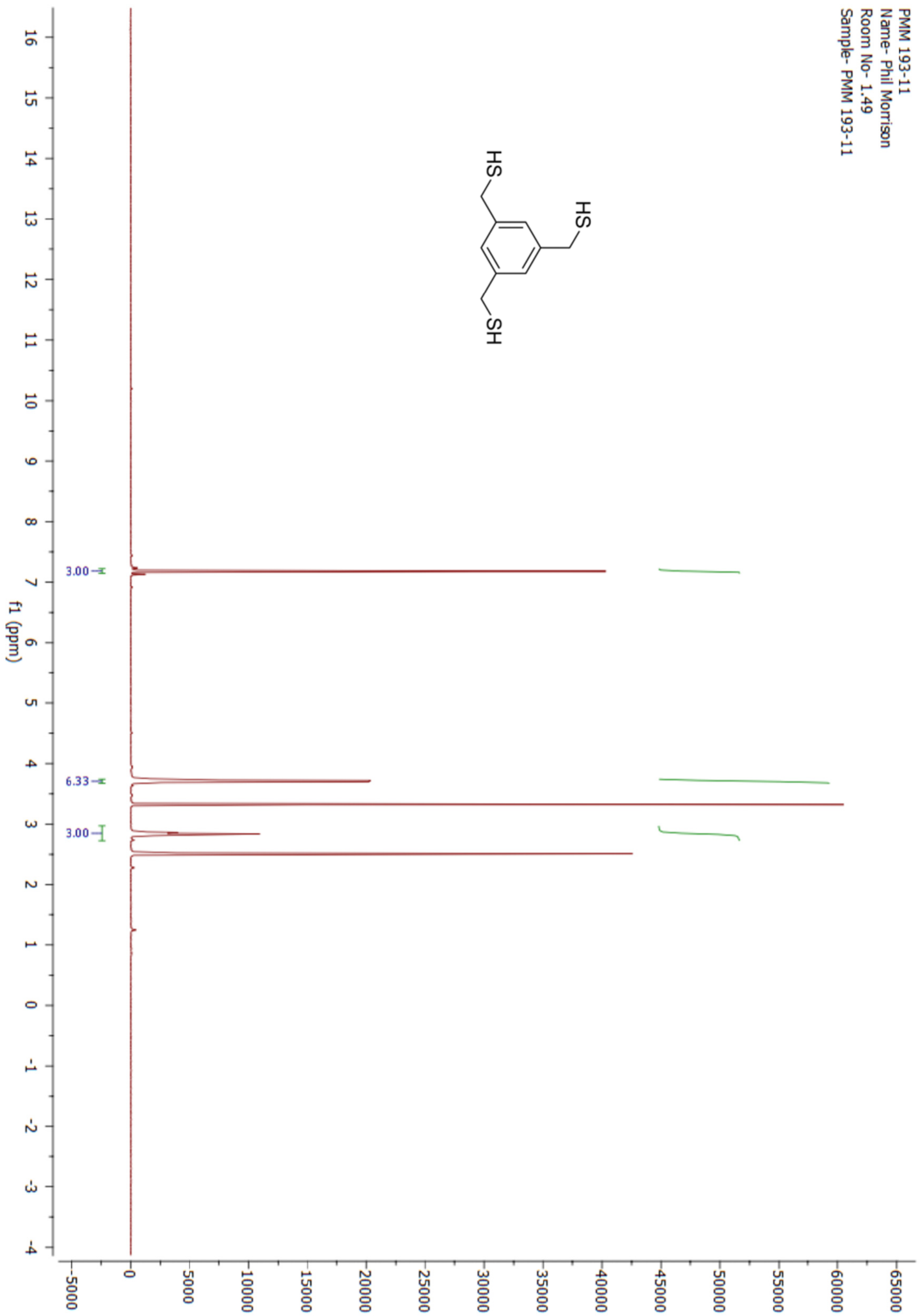

[1] J. M. Chalker, S. B. Gunnoo, O. Boutureira, S. C. Gerstberger, M. Fernández-González, G. J. L. Bernardes, L. Griffin, H. Hailu, C. J. Schofield, B. G. Davis, *Chem. Sci.* **2011**, *2*, 1666.
[2] C. Heinis, T. Rutherford, S. Freund, G. Winter, *Nat. Chem. Biol.* **2009**, *5*, 502-507.
